# Genetic variants analysis of three dromedary camels using whole genome sequencing data

**DOI:** 10.1371/journal.pone.0204028

**Published:** 2018-09-20

**Authors:** Reza Khalkhali-Evrigh, Seyed Hasan Hafezian, Nemat Hedayat-Evrigh, Ayoub Farhadi, Mohammad Reza Bakhtiarizadeh

**Affiliations:** 1 Department of Animal Breeding and Genetics, Sari Agricultural Sciences and Natural Resources University, Sari, Iran; 2 Department of Animal Science, Faculty of Agriculture and Natural Resources, University of Mohaghegh Ardabili, Ardabil, Iran; 3 Department of Animal and Poultry Science, College of Aburaihan, University of Tehran, Pakdasht, Iran; University of Lausanne, SWITZERLAND

## Abstract

Whole genome wide identification and annotation of genetic variations in camels is in its first steps. The aim of this study was the identification of genome wide variants, functional annotations of them and enrichment analysis of affected genes using whole genome sequencing data of three dromedary camels. The genomes of two Iranian female dromedary camels that mostly used to produce meat and milk were sequenced to 41.9-fold and 38.6-fold coverage. A total of 4,727,238 single-nucleotide polymorphisms (SNPs) and 692,908 indels (insertions and deletions) were found by mapping raw reads to the dromedary reference assembly (GenBank Accession: GCA_000767585.1). In-silico functional annotation of the discovered variants in under study samples revealed that most SNPs (2,305,738; 48.78%) and indels (339,756; 49.03%) were located in intergenic regions. A comparison of the identified SNPs with those of the African camel (BioProject Accession: PRJNA269274) indicated that they had 993,474 SNPs in common. We found 15,168 non-synonymous SNPs in the shared variants of the three camels that could affect gene function and protein structure. Obtained results revealed that there were 7085, 6271 and 4688 non-synonymous SNPs among the 3436, 3058 and 2882 genes in the specific gene sets of Yazd dromedary, Trod dromedary and African dromedary, respectively. The list of genes predicted to be affected by non-synonymous variants in different individuals was subjected to gene ontology (GO) enrichment analysis.

## Introduction

Based on the palaeontological evidence, the Camelidae family appeared for the first time in North America in 45.9 Mya [1]. There are at present two domesticated camels globally, the dromedary (one-humped) and Bactrian (two-humped) camels, which belong to the genus *Camelus*. The division between dromedary and Bactrian camels occurred about 4.4 Mya during the migration of camel ancestors from North America to Eurasia [2].

There are more than 27.7 million camels globally [3]. They are used for food and racing in many Northern African and Asian countries. About 90% of extant camels are dromedaries [4], which are best known for their unique ability to survive in harsh desert environments. Dromedary camels in most regions of Asia and Africa are used as pack animals and as a source of food for nomads and low income rural populations. Their unique ability to withstand harsh environmental conditions coupled with their medical benefits [5–8], make camels an ideal case for further studies.

Because camels are generally raised in developing countries, studies on them are limited when compared with popular domesticated animals (cattle, sheep, pigs, horses). Draft genome sequences recently have been made available for dromedary [2,9] and Bactrian [10] camels, but they have not been assembled into chromosomes at this time.

To the best of our knowledge, no study has been published on whole genome resequencing of native Iranian camels. Over 84% of Iran is arid or semi-arid [11]. Some areas have been influenced by increasing population pressure on land and water resources and are at risk of desertification [12]. Climatic conditions, along with traditional religious and cultural values of Iranians represent a high potential for camel breeding. Unfortunately, camel breeding in Iran, as in most parts of the world, is not done scientifically. Nonetheless, it appears that whole genome resequencing of the native Iranian camel genome could increase understanding of the evolution of the camel [2] and provide a valuable opportunity for breeding programs.

The concept of genomic selection proposed by Meuwissen et al. (2001) [13], as well as the advent and improvement of next-generation sequencing technology, has revolutionized the use of genetic information for livestock breeding programs. Implementation of genomic selection for dairy cattle [14], sheep [15] and pig [16] breeding has produced promising results in terms increasing the rate of genetic improvement. The determination of large-scale genetic variation, especially SNP markers, using whole genome sequencing could lead to development of approaches such as genome-wide association studies and genomic selection in camel breeding.

Despite recent studies on camels at the genomic level, a large number of genetic variants in different camel breeds remain to be discovered and properly annotated. The present study reports on the first whole genome resequencing of individual camels from two distinct geographical regions, Trod station located in Semnan province and Yazd station located in Yazd province in Iran. In fact, sampled camels from Trod and Yazd stations considered as genetic representatives of camels that live in northern half (e.g. Golestan, Semnan and Qom) and southern half Provinces (e.g. Kerman, Fars and Hormozgan) of Iran, respectively. In order to identify the SNPs and indels (insertions and deletions), the paired-end reads of each sequenced individual were mapped to the dromedary camel reference genome (GenBank Accession: GCA_000767585.1). In the next step, in-silico functional annotation was carried out for the identified dromedary genomic variants. In addition, to investigate relationships between Iranian and African dromedaries, SNP discovery analysis was carried out using previously-reported whole genome sequences of the African dromedary (BioProject Accession: PRJNA269274) as a non-Iranian genetic resource.

## Materials and methods

### Sampling and DNA extraction

In the present work, we sequenced the genomes of a female camel obtained from Trod station in Semnan Province (TrD) and a female camel obtained from Yazd station in Yazd Province (YaD). Blood samples were collected from the jugular vein using 4 ml vacutainer tubes and stored at -20C° until use. To reduce stress of animals, positive rewards such as petting was implemented in the conditioning to regular handling prior to restraint for blood collection. All animal care and experiments were approved by the animal science committee of the University of Mohaghegh Ardabili, Iran. Also, all experiments were performed in accordance with a routine guideline which is acceptable by this committee.

DNA extraction was performed using a RBC mini kit for mammalian blood following manufacturer protocols (Real Biotech; South Korea). The extracted DNA was quantified using NanoDrop and the 260/280 ratios identified were 1.90 and 1.8 for YaD and TrD, respectively. The quality of the DNA samples was assessed using gel electrophoresis in 1% agarose gel. A library with an average insert size of ~360 bp was generated and two lanes of 100 bp paired-end sequencing was carried out using the Illumina HiSeq 2000 system (Illumina, San Diego, CA).

### Mapping short reads and variant calling

Quality control of the raw sequencing reads was performed using FastQC (http://www.bioinformatics.bbsrc.ac.uk/projects/fastqc/). Quality filtering of the short reads was carried out using the maximum information (MaxInfo) approach of Trimmomatic [17] version 0.36, with a target length of 40 and strictness value of 0.5. Reads with a length of less than 40 bp were discarded. The MaxInfo algorithm performs an adaptive trim that creates a balance between the benefits of maintaining longer reads against the value of maintaining bases with errors. Clean reads were mapped to the dromedary camel genome assembly (GenBank Accession: GCA_000767585.1; version: PRJNA234474_Ca_dromedarius_V1.0) as a reference using Burrows Wheeler aligner (version 0.7.15; BWA-MEM algorithm) [18]. Picard tools (http://broadinstitute.github.io/picard) were then used to remove the duplicated reads. Duplicated reads were considered to be identical reads that arise during PCR and are mapped to same genomic position during mapping to the reference [19].

Local realignment around indels was carried out using the Genome Analysis Toolkit (GATK) [20] in two steps to enhance mapping quality. In the first step, RealignerTargetCreator module determines the intervals to target for local realignment and realigning over those intervals was carried out using the IndelRealigner command. Base quality scoring recalibration (BQSR) was applied to obtain more accurate base qualities using GATK. It is worth to mention that, due to lack of sufficient genomics data for camels (such as known variants), we used resulted VCF file after local realignment step as known VCF in BQSR step. Putative variant calls were made using GATK (and the HaplotypeCaller algorithm) and samtools [21] mpileup. All variants were identified as differences from the reference genome. To obtain reliable variants, the overlap variants between the outputs (VCF files) of the two mentioned variant callers were extracted using BEDTools. Variants with phred-scaled scores below 20 and variants with genotypic qualities (GQ) of less than 20, SNPs within 5 bp of an indel, indels within 10 bp of each other, variants with a depth of coverage below 33% or more than twice mean genome coverage of the alignment were removed to generate a final variants list for downstream analysis. The program VCFtools [22] (version 0.1.13) was used to calculate the transition-to-transversion (Ti/Tv) ratio as a parameter to assess the specificity of new SNP calls.

### Variant annotation

SnpEff [23] was used to assign the impacts of the variants and their functional grouping. Because there is no database for dromedary camels among the pre-built databases for SnpEff, we built a dromedary database using the dromedary reference genome and its GFF (version 3) file using the database building guidelines (http://snpeff.sourceforge.net/SnpEff_manual.html#databases) in SnpEff.

### Functional enrichment analysis

The list of genes predicted to be affected by non-synonymous variants in different individuals was subjected to gene ontology (GO) enrichment analysis using the database for Annotation, Visualization and Integrated Discovery (DAVID) [24] version 6.8. For each gene set, the genes were sorted based on number of their non-synonymous SNPs and the 1000 first genes with highest number of mutations were selected for further analysis. The calculated p-values were corrected using the Benjamini correction for multiple testing and enriched terms were considered statistically significant at p-adjusted < 0.1. It is worth to mention that, we investigated loss-of-function (LOF) variants in shared variants (among three samples) set and their enrichment analysis.

### Comparison of Iranian and African dromedaries

The samples were compared with the geographically-distinct dromedary using raw sequence data for the domestic dromedary of North African origin (trimmed and error-corrected paired-end reads; accession no. SRX1013838). The ancestry of the African dromedary (AfD) can be traced to the Canary Islands [9]. Because downloaded paired-end reads were corrected and trimmed previously by Fitak et al (2016) [9], the trimming step was not carried out for this dataset. Reference mapping, processing after alignment, variant calling and another analyses for the downloaded sample were performed according to the workflow described above.

## Results and discussion

### Sequencing and mapping to reference

The present study reports on the first whole genome resequencing of two female native camels (YaD and TrD samples) as samples of dromedaries originating from Asia and characterized their genetic variations, including SNPs and indels. Whole genome resequencing of the YaD and TrD samples produced 920,366,954 and 843,455,144 paired-end reads with a read length of 100 bp, respectively. A total of 899,714,102 and 826,229,484 reads remained after filtering the YaD and TrD samples, respectively (Table 1). It is well documented that variant calling mainly depends on data coverage. It has been reported that, for high-coverage data, variant discovery led to a lower number of false positives than for a low-coverage data set [25]. Here, high-coverage data was generated to improve the results.

**Table 1:** Summary of sequenced reads for Iranian dromedaries and downloaded sample (AfD).

	Total Reads		Mapped		
Genome	Before trim	After trim	Reads	Bases	Fold coverage
**YaD**	920,366,954	899,714,102	879,503,046	83,966,061,113	41.9
**TrD**	843,455,144	826,229,484	804,795,688	77,257,841,038	38.6
**AfD**	-	1,124,832,578	1,112,171,199	107,907,751,223	53.8

Approximately 97.8% and 97.4% of clean reads for YaD and TrD, respectively, were mapped to the reference dromedary camel genome and indicate that the resequencing data covered most of the genome. Successfully-mapped reads yielded 41.9-fold and 38.6-fold coverage for YaD and TrD, respectively (Table 1). Fold coverage was calculated by dividing the successfully-mapped bases by the length of the assembled reference genome used for mapping (2,004,047,047 bp). Similar to this method, the genome of AfD previously had been sequenced using the Illumina HiSeq 2000 platform [9]. The percentage of successfully-mapped reads to reference (98.9%) and the fold coverage of AfD represented higher values than for Iranian camels (Table 1). In comparison with whole genome resequencing studies in cattle [26–28], horse [29] and chickens [30] performed to variant identification, the current study obtained a higher depth of coverage, which made a significant improvement in the accuracy and sensitivity of variant calling [31].

### SNP identification

It is known that the accuracy of variant discovery and the detection rate is dependent on the different variant discovery algorithms. Two widely-used variant discovery tools were used on the samples to minimize false positive results and optimize the pipeline. The higher accuracy and sensitivity of these tools has been reported in previous studies [32]. The number of identified SNPs for the YaD, TrD and AfD camels, based on the intersection of two SNP calling and filtering algorithms, were 2.4, 2.3, 2.1 million variants, respectively. It is well-documented that the number of variants detected depends mainly on the sequencing depth and that an increase in sequence depth significantly improves both the accuracy and sensitivity of variant discovery [32].

**Fig 1:**
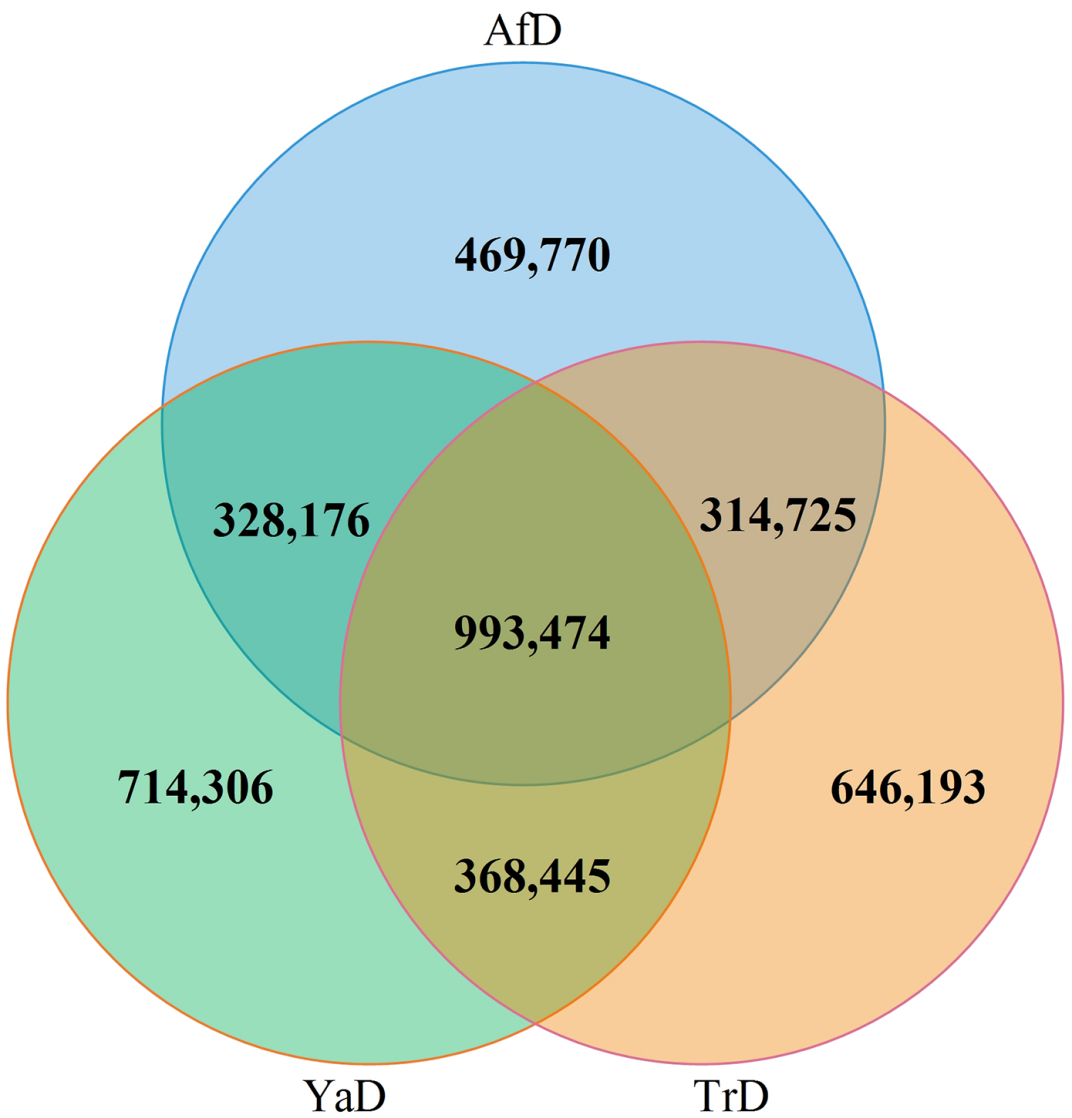
Overlapping and sample specific identified SNPs in Iranian dromedaries (YaD and TrD) and downloaded sample (AfD).

Of the 6,833,383 SNPs identified in the three camels, 993,474 SNPs were shared across all individuals, with 491,851 and 501,623 being heterozygous and homozygous, respectively. Approximately 56.6% of SNPs from the YaD variant set and 58.6% of SNPs from the TrD variant set were in common (1,361,919 SNPs). The considerable number of unique SNPs identified for the Iranian dromedaries indicates the importance of resequencing to identify novel variants for investigating genetic diversity in the different camel populations. The results shown in Fig 1 illustrate the high number of shared SNPs between Iranian dromedaries and AfD, such that 54.9% (1,321,650) and 56.3% (1,308,199) of identified SNPs in YaD and TrD were common with the SNP set of AfD. The density of SNPs was determined to be approximately one per 833, 863 and 952 bp for YaD, TrD and AfD, respectively. The mutational frequency was assessed at the single nucleotide level in the three samples. The results revealed that the dominant mutation type was G to A (and C to T after non-strand specific DNA-Seq data was applied) among all camels. This includes 854408 (35.54%), 823846 (35.46%) and 746682 (35.45%) for YaD, TrD and AfD, respectively (S1 Table).

SNP quality was further assessed by calculating the Ti/Tv ratio for each SNP set, because this measurement is used as an indicator of potential sequencing error [33]. Ti/Tv ratio in our final sets of SNPs was 2.34, 2.34 and 2.33 for YaD, TrD and AfD, respectively. The results of Ti/Tv ratio in the current study was in accordance with previous findings for the dromedary camel (2.31) [9], but slightly higher than for that of Korean native cattle (2.24 for Hanwoo and Jeju Heugu breeds) [28]. Higher Ti/Tv ratio (as quality evaluator of detected variants) in our study may represent good quality of detected SNPs.

Among the camels under study, YaD had highest number of heterozygous SNPs (1,659,085), whereas AfD with 1,375,721 heterozygous SNPs ranked third (Table 2). Because the number of total SNPs differed among the three camels, it appears that calculation of the percentage of heterozygous SNPs from among the total SNPs, allows clear comparison of the heterozygosity rate in the individuals. Of the total identified SNPs in the Iranian camel, 69% (YaD) and 68.4% (TrD) corresponded to heterozygous SNPs and were higher than for AfD with 65.3% heterozygosity. Higher heterozygosity in Iranian camels can most likely be explained by the rearing system and lack of organized breeding schemes for camels in Iran. Each spring, Iranian herders in central regions, after shearing, treating and selection of young camels for sale, leave their camel herds loose in the desert for the rest of year. Population mixture and free mating between camels under such a rearing system increases gene flow and heterozygosity. Of course, to prove this, more re-sequenced samples are required. Another reason for higher heterozygosity in Iranian dromedaries can be due to mating with Bactrian camels that live in Iran. We found that Bactrian camels have introgression into Iranian dromedaries based on 20 microsatellite data studied by Hedayat et al (2018) [34].

**Table 2:** Summary of identified variants for Iranian dromedaries and downloaded sample (AfD).

	YaD	TrD	AfD
**Number of SNPs**	2,404,401	2,322,837	2,106,145
**TS/TV**	2.34	2.34	2.33
**Heterozygote SNPs**	1,659,085	1,588,757	1,375,721
**SNP Het/Hom**	2.23	2.16	1.88
**Insertion**	186,185	181,306	179,801
**Deletion**	165,244	160,173	154,473
**INDEL/SNP**	0.15	0.15	0.16

### Functional annotation of detected SNPs

Here, functional annotation of the identified variants was carried out using SnpEff and they were classified into seven locational (e.g., intergenic, intron and intragenic) and eight functional categories (e.g., stop_gained, start_lost and non_synonymous_coding). Approximately 87.8% of detected SNPs in all three samples were located in intergenic and intron regions (Table 3). These SNPs have modifier impact and the prediction of their effect on phenotype is difficult. Moreover, of all discovered SNPs, 160,238 were located in coding regions. We found that only 0.11% (2,773 SNPs for YaD, 2,608 SNPs for TrD and 2,414 SNPs for AfD) of SNPs had a high impact on the products of genes and probably cause structural changes in the respective proteins.

**Table 3:** Functional annotation of discovered SNPs.

Impact	SNPs	YaD	TrD	AfD	Shared SNPs
**HIGH**	STOP_GAINED	1343	1271	1217	486
	STOP_LOST	979	922	838	472
	START_LOST	124	111	98	51
	SPILICE_SITE_ACCEPTOR	127	116	103	54
	SPILICE_SITE_DONOR	200	188	158	93
**MODERATE**	NON_SYNONYMOUS_CODING	36748	35208	31506	15168
**LOW**	SYNONYMOUS_STOP	181	151	154	77
	SYNONYMOUS_START	1	-	-	-
	NON_SYNONYMOUS_START	10	11	9	5
	SYNONYMOUS_CODING	17310	16354	14780	7001
**MODIFIER**	INTERGENIC	1164972	1140766	1036643	486742
	INTRAGENIC	8	8	4	1
	INTRON	943845	899719	815981	386512
	UPSTREAM	132089	125375	112771	53393
	DOWNSTREAM	106464	102637	91883	43419
	TOTAL	2404401	2322837	2106145	993474

Non-synonymous SNPs, also called missense variants, led to changes in protein effectiveness due to changes in amino acid codons. Out of all non-synonymous SNPs discovered in the three camels, there were 15,168 SNPs in common between them. Furthermore, it was found that 7085, 6271 and 4688 non-synonymous SNPs among 3436, 3058 and 2882 genes were specific for YaD, TrD and AfD, respectively, which provides valuable resources to be used in genetic analysis of the phenotypic differences among them. GO analysis was performed for the genes, including specific non-synonymous SNPs in each individual to investigate whether these genes are associated with any biological terms. Functional enrichment analysis was carried out for genes containing non-synonymous SNPs shared among three camels. The first 1000 genes based on the number of non-synonymous SNPs from each gene list were extracted and functional enrichment analysis was carried out on them (S2–S5 Tables). The range of non-synonymous SNPs for selected genes to Go analysis for shared SNPs set, YaD-specific set, TrD-specific set and AfD-specific set were, 2–29, 2–32, 2–25 and 1–20, respectively.

The results showed that there was no significant over-represented GO t-erm for biological processes in the YaD-specific gene list. However, significant results were found for cellular components and molecular function. Significantly enriched GO terms for TrD-specific, AfD-specific and shared genes were also observed. One of the most interesting enriched term in the shared genes set was “cellular response to osmotic stress” (GO:0071470), which could be vital for animals and plants that live in a desert environment. Some biological processes, such as DNA and protein damage, prevention of DNA replication and transcription, inhibition of protein production and mitochondrial depolarization can occur due to osmotic stress [35]. The high salt concentration in a camel diet and water shortage in deserts mean that camels are highly exposed to osmotic shock. Presumably, our finding indicates that some genes such as those found in the GO analysis have raised camel tolerance to high salt levels [10] and hyperosmotic stress.

It was found that microtubule-related terms were common to shared genes, and TrD-specific and AfD-specific sets. The GO term “microtubule-based process” is any cellular process that depends upon or alters the microtubule cytoskeleton, that part of the cytoskeleton comprising microtubules and their associated proteins. A microtubule-based movement is a process that results in the movement of organelles, other microtubules or other cellular components. An “actin filament-based process” is as any cellular process that depends upon or alters the actin cytoskeleton, that part of the cytoskeleton comprising actin filaments and their associated proteins [36]. Enrichment of these terms may be related to the role of the cytoskeleton in renal development [37].

The kidney is a very important organ in the body of camels because of its high urine-concentrating and water reabsorption capacity, which help the camel survive in the arid conditions of hot deserts. Microtubules are involved in ultrafiltration of plasma by the glomerulus, in water and solute reabsorption and in the secretion of anions and cations into tubular fluid [38]. These functions enable the kidney to adjust the volume and composition of urea and are critical for the camel. High NaCl and ultraviolet radiation are significant features of deserts that increase DNA damage [35]. Investigation of molecular function among the enriched gene sets revealed that enriched terms such as “nucleoside binding” (GO:0001882), “ATP binding” (GO:0005524) and “kinase activity” (GO:0016301) could be involved in DNA repair and replication.

Of the 324 positively-selected genes (PSG) identified by Wu et al. (2014) [2] in the dromedary camel, we found 96, 43, 47 and 40 PSGs in the shared, YaD-specific, TrD-specific and AfD-specific gene sets, respectively (S6 Table). A large portion of PSGs in genes (containing non-synonymous mutation) shared among camels over long geographical distances (Iran and Africa) can be attributed to fixation of these mutations in dromedaries, although the proof of this claim requires further resequencing of different breeds of dromedary camels globally.

Dense dust in deserts puts camels at high risk of respiratory diseases, but it appears that evolution has provided camels with the ability to deal with this threat. Wu et al. (2014) [2] reported several genes, including FOXP3, CX3CR1, CYSLTR2 and SEMA4A, that are related to respiratory diseases as PSGs in dromedary and Bactrian camels. Interestingly, annotation of identified SNPs in variant sets shared among the three camels revealed that 2, 2 and 1 non-synonymous SNPs occurred in FOXP3, CYSLTR2 and CX3CR1, respectively. In light of these results, the importance of genes associated with respiratory system of camels is clear.

It has been reported that the fixed SNPs probably represent alleles present at the time of domestication [39]. In the current study, fixed SNPs are the positions where there were homozygous SNPs in Iranian camels, but AfD had none of these SNPs in those positions. Of the 101,433 identified SNPs with the mentioned criteria, 1,987 missense SNPs were detected as fixed SNPs and were located in 837 genes. Because these variants were found only in the Iranian camel, this may indicate that the variants in these regions became fixed by human selection after domestication and can be considered alleles in diversification or improvement genes. To further investigate the differences between the breeds, functional enrichment analysis was performed on the genes that contained fixed SNPs. This analysis revealed that genes related to cell adhesion and biological adhesion were over-represented in the set of fixed SNPs for Iranian camels. Interestingly, some fixed SNPs were located in LDLR, MLYCD, APOE and PPARGC1A genes involved in lipid and energy metabolism. Reservation and utilization of hump fat is a routine process throughout camel life; thus, optimal metabolism of lipids is a key factor for enduring food and water shortages.

Investigation of LOF variants revealed that there are 439 LOF variant in shared variants set. Of the identified SNPs, 197 and 242 SNPs were heterozygous and homozygous, respectively. Because the camel genome project is in its early stages and genomic information in this field is not complete, the downloaded GFF file has not yet been completed and the name of a number of genes is unknown. After removing of unknown genes, enrichment analysis was performed for the remaining 352 genes. We observed no significantly enriched biological process GO term for mentioned genes.

### GO enrichment of specific Iranian camel genes

It was found that 1,874 genes containing non-synonymous SNPs were specific for Iranian samples when compared with AfD. GO enrichment analysis of these genes produced 45 enriched terms at an un-adjusted p-value ≤ 0.01 (S7 Table). Investigation of the results revealed that 15 (33.3%) enriched terms (such as “nephron development”, “kidney morphogenesis” and “renal tubule development”) are related to morphogenesis and development of the urinary system, especially the kidneys. It was also observed that eight (17.8%) terms (such as “regulation of T- cell activation”, “regulation of type I interferon production” and “regulation of lymphocyte activation”) are directly related to immune response. The combination of immune-related terms with “regulation of response to stimulus” (GO:0048583), “response to mechanical stimulus” (GO:0009612), “regulation of response to stress” (GO:0080134), “regulation of hemostasis” (GO:1900046) and “wound healing” (GO:0042060) may be reflects the successful adaptation of Iranian dromedaries to living under the stressful conditions of the central deserts of Iran.

### Indel identification

Indels are structural variants that have significant effect on gene structure, expression and function [40]. Next-generation sequencing has generated a unique opportunity to indel identification on the large scale. In our resequencing project, the total number of identified indels across the genomes of the three camels was 1,027,182, of which 53.3% (547,292) were insertions. A total of 133,274 indels were common to all three camels while the indels with no overlap with any other sample represented 112,824 in YaD, 104,391 in TrD and 96,915 in AfD (S1 Fig). Among the three camels, YaD had largest number of indels (351,429), as was in accordance with a large number of SNPs in this camel. These results indicated that YaD, in comparison with other samples, was more divergent than the reference camel genome. The length of the detected indels ranged from +30 (insertion) to -45 (deletion) base pair. The results revealed that 1–3 bp indels were 66.5%, 65.9% and 64.7% of all discovered indels in YaD, TrD and AfD, respectively (S2 Fig).

### Functional annotation of detected indels

**Fig 2:**
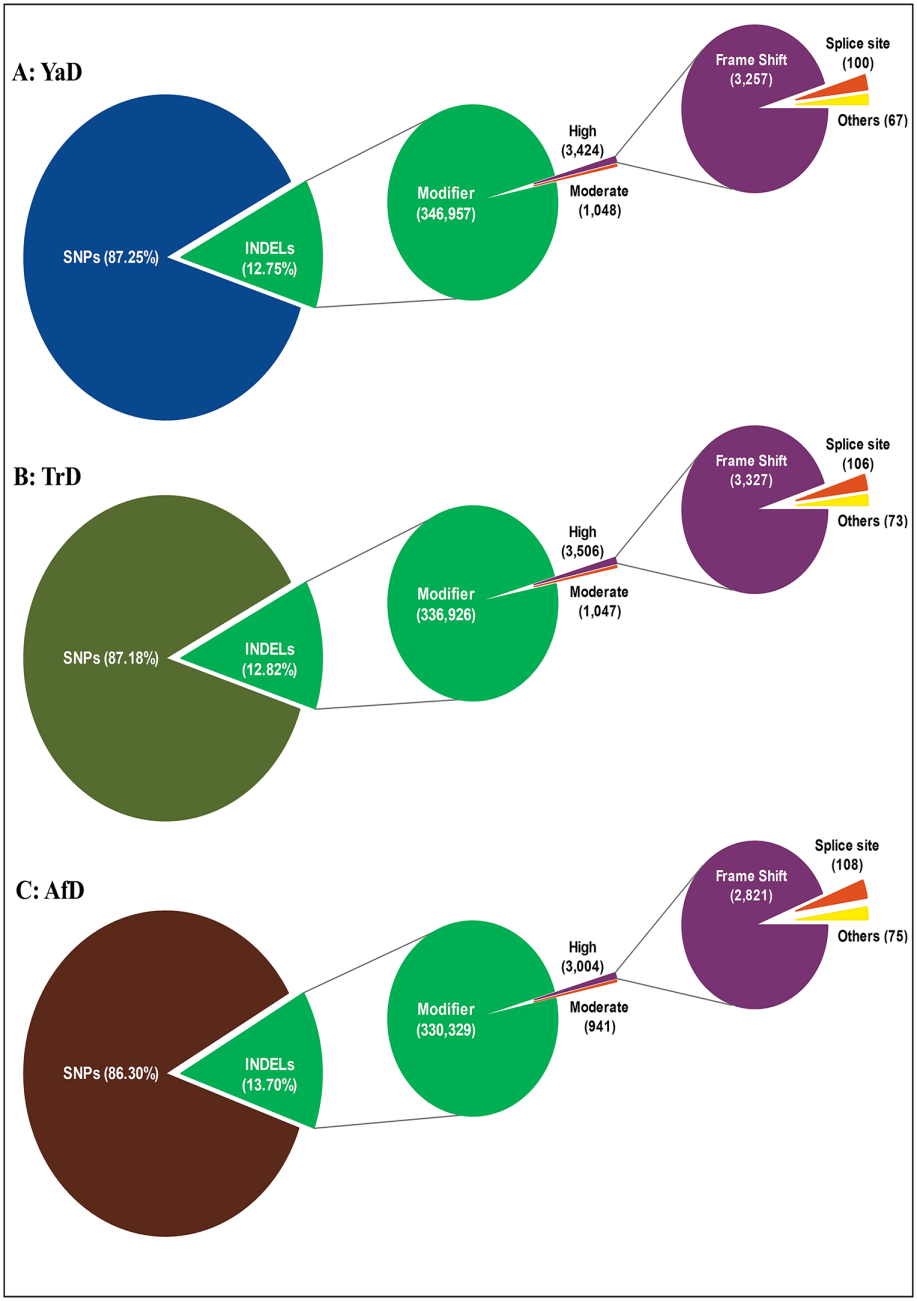
Classification of identified indels by their impact on genome for YaD (A), TrD (B) and AfD (C).

A summary of functional annotation for Iranian dromedaries and the downloaded sample (AfD) is shown in Table 4. The majority of annotated indels in the three camels are located in the intergenic and intron region (913,342; 88.9% of all indels). It was found that the 3257 (0.93%), 3327 (0.97%) and 2821 (0.84%) indels for YaD, TrD and AfD, respectively, cause a frameshift event (Fig 2). Frameshift mutations are classified in the important disruptive mutation group and change the reading frame of protein coding sequences [41]. The percentage of frameshift mutations in the camel genome was higher than in the cattle genome (0.08%) [27–28], but lower than in the horse genome (1.3%) [29]. Of the three discovered indel sets, 9934 (0.97%), 3036 (0.30%) and 1014212 (98.73%) indels were grouped in variants having a high effect, moderate effect and modifier effect, respectively (Fig 2). Gene fusion (four indels) and gene fusion reverse (three indels) classes had the lowest numbers of indels among variants with a high effect. All seven indels that caused gene fusion and gene fusion reverse were deletions. The lengths of the gene fusion indels in YaD were 13 and 32 bp, while there were 25 and 32 bp indels in this class for TrD.

**Table 4:** Functional annotation of discovered indels.

indel	YaD	TrD	AfD
**INTERGENIC**	171706	168050	165086
**INTRON**	140774	135017	132709
**UPSTREAM**	19030	18577	17898
**DOWNSTREAM**	15445	15280	14636
**EXON**	4371	4446	3836
**SPILICE_SITE_ACCEPTOR**	55	53	58
**SPILICE_SITE_DONOR**	45	53	50
**GENE_FUSION**	2	2	-
**GENE_FUSION_REVERSE**	1	1	1

## Conclusion

In the current study, whole genome resequencing of two Iranian dromedary camels was carried out. The raw reads from the Illumina Hiseq system were mapped to the reference genome and variants were identified using two powerful variant callers (SAMtools and GATK). Overall, 4,727,238 SNPs and 692,908 indels were identified for the two Iranian camels. The variants were annotated and classified into functional and locational categories. The whole genome resequencing project provides a valuable resource for future studies. Increasing genome-wide information in camels could improve understanding about camel breeding (e.g; genomic selection).

We tried to use our whole genome sequencing data along with publicly available genomic data of camels for genetic variants analysis of two Iranian dromedary camels and one African origin camel, which leads to identify Go categories related to survival in harsh condition of deserts. However, the main issue for confirmation of this study was the number of samples. The big challenge in research on camels, especially at the genomic level, is the shortage of reliable sources to receive scientific information. It appears that researchers must focus on enriching the genome-level information in this field. We took one step (one of the first steps) on re-sequencing analysis of camels and the obtained results along with another parallel projects could help to enrich the information resources associated with camels.

## Supporting information

S1 Fig Overlapping and sample specific identified indels in Iranian dromedaries (YaD and TrD) and downloaded sample (AfD). (TIF)

S2 Fig Distribution of all identified indels size in YaD, TrD and AfD. (TIF)

S1 Table Frequency of single nucleotide mutations in identified SNPs for YaD, TrD, AfD and shaired variants among three sets, respectively. (XLSX)

S2 Table Results of GO enrichment analysis for YaD-specific genes set. (XLSX)

S3 Table Results of GO enrichment analysis for TrD-specific genes set. (XLSX)

S4 Table Results of GO enrichment analysis for AfD-specific genes set. (XLSX)

S5 Table Results of GO enrichment analysis for shared genes set (among YaD, TrD and AfD). (XLSX)

S6 Table PSGs containing non-synonymous SNPs in YaD, TrD, AfD and shared gene sets. (XLSX)

S7 Table Results of GO enrichment analysis for Iranian camel specific genes. (XLSX)
