# Supplementary figures and images for: Genetic variants analysis of three dromedary camels using whole genome sequencing data
Source: PLoS One. 2018 Sep 20;13(9):e0204028. doi: 10.1371/journal.pone.0204028 (PMC6147446; doi:10.1371/journal.pone.0204028)

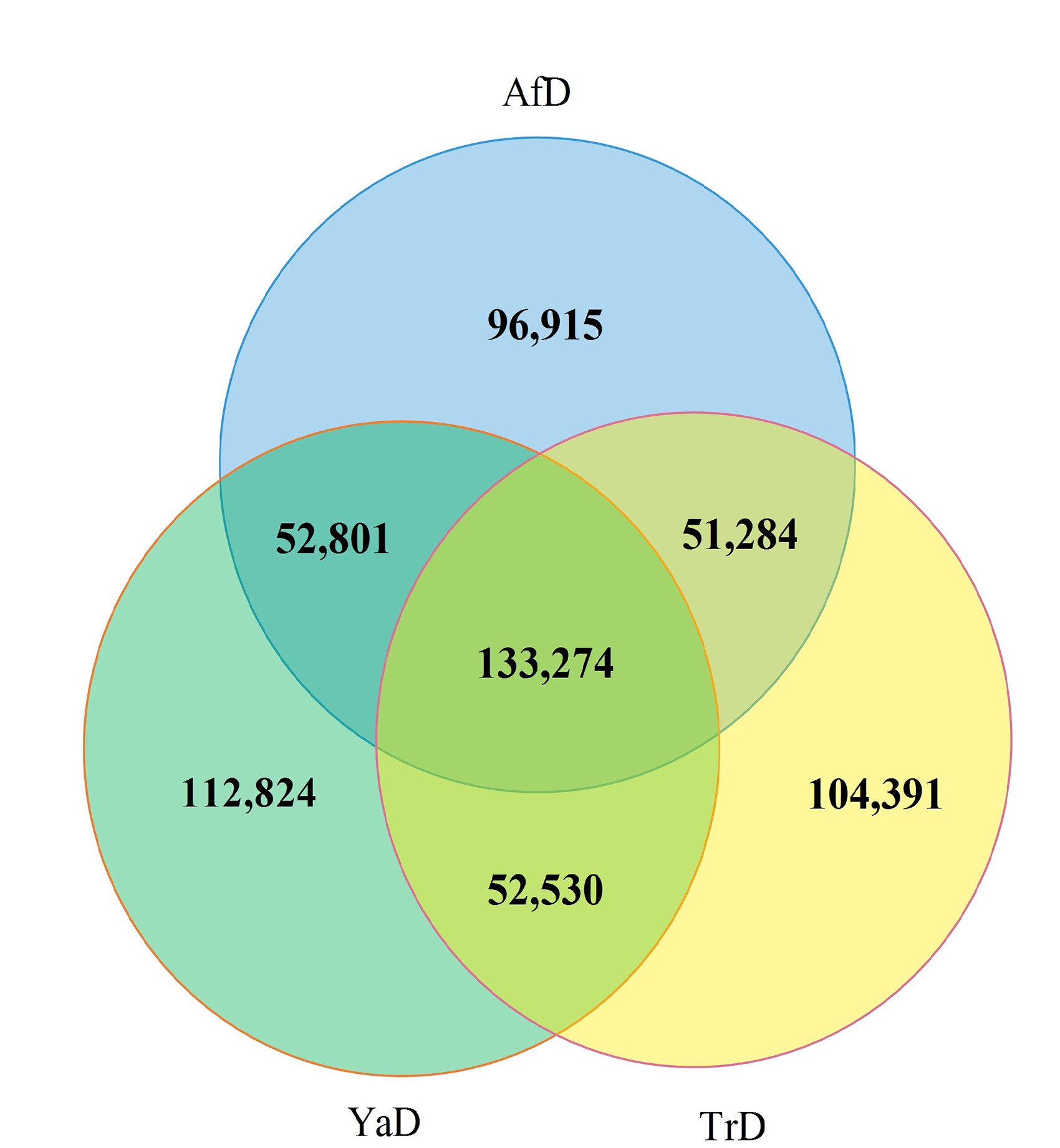

Supplement: S1 Fig — (TIF) [file pone.0204028.s001.tif]

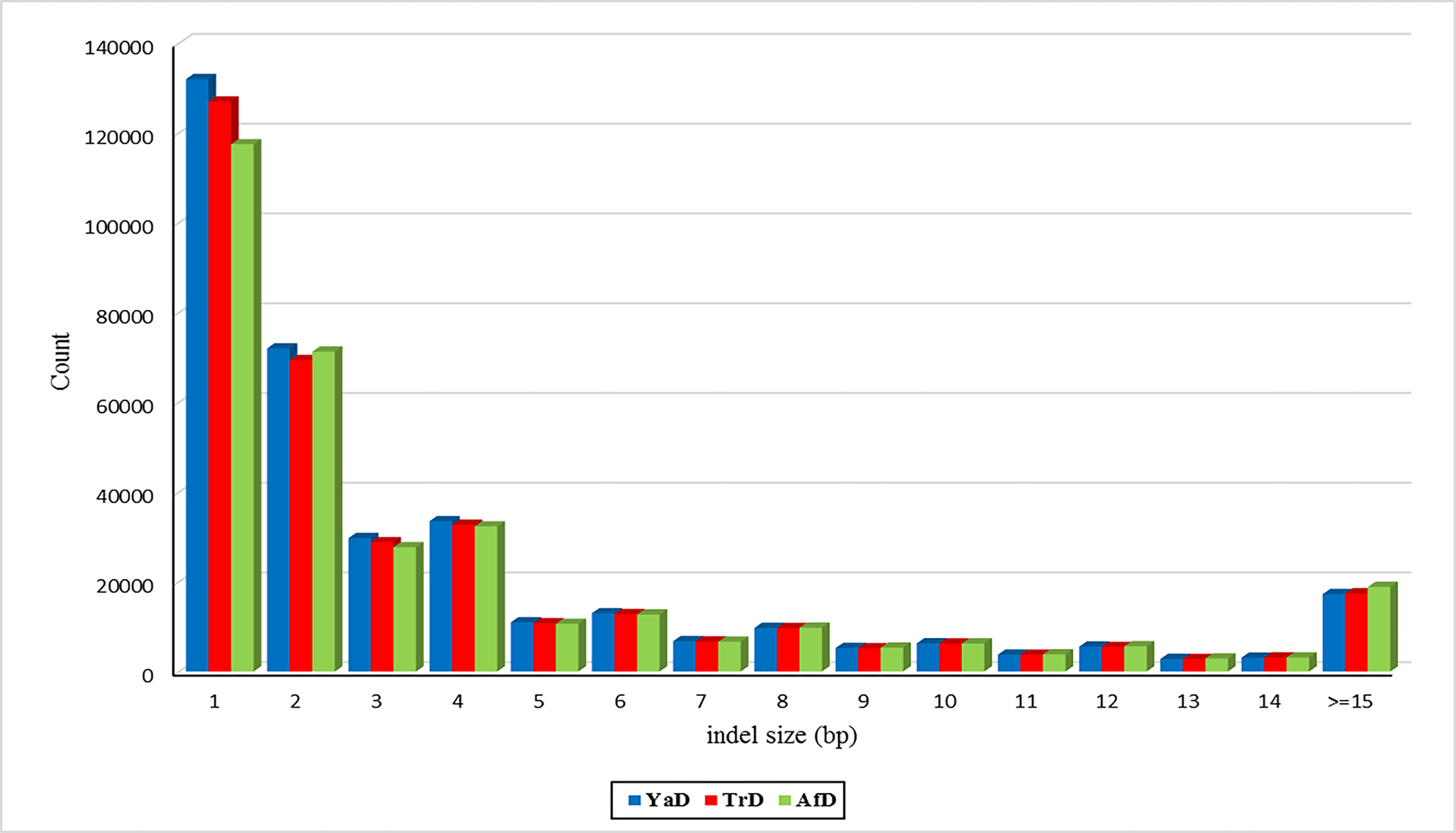

Supplement: S2 Fig — (TIF) [file pone.0204028.s002.tif]
